# Iron’s True Weight: Does the Amount of Iron in the Body Equate to the Amount of Iron on the Bar in Australian Football League Women’s Players?

**DOI:** 10.3390/nu17101691

**Published:** 2025-05-16

**Authors:** Michael Pengelly, Kate L. Pumpa, David B. Pyne, Naroa Etxebarria

**Affiliations:** 1 Research Institute for Sport and Exercise (UCRISE), University of Canberra, Canberra, ACT 2617, Australia; kate.pumpa@ucd.ie (K.L.P.); david.pyne@canberra.edu.au (D.B.P.); naroa.etxebarria@canberra.edu.au (N.E.); 2 Institute for Sport and Health, University College Dublin, Belfield, D04 C1P1 Dublin, Ireland

**Keywords:** women, mineral deficiency, training, strength, power, performance

## Abstract

Background: The physiological requirements of a successful team sport performance partly depend on iron-facilitated mechanisms. However, how low iron stores affect team sport athletes remains unclear. Purpose: To explore the influence of iron status on strength and power performance in elite female Australian Rules Football players. Methods: Iron indices were measured in 30 players (age 23 ± 4 y; body mass 70 ± 6 kg) at the start and end of the 10-week preseason. Players were categorized as iron deficient (ID; serum ferritin (sFer) < 40 µg/L) or iron sufficient (sFer > 40 µg/L). Over this period, three-repetition maximum and sport-specific performance measures were evaluated. Results: Approximately 80% of all the sFer samples primarily ranged between 9 and 60 µg/L. Strength (e.g., squat, bench press) was up to 13% lower in ID players in week 1, with no substantial differences between groups during week 10. There were marginal differences (ID: −1% to +3%) in performance for all the remaining measures between groups (e.g., 10-m sprint). Very weak to moderate correlations were observed between all the performance measures and fixed effects (e.g., sFer, other strength assessments), increasing to moderate to very strong correlations when accounting for random effects (athlete). Conclusions: Iron deficiency may compromise strength performance, but this shortcoming may not translate to measures of power and speed. Individualized iron monitoring practices for athlete health and performance are encouraged.

## 1. Introduction

Iron is an essential micronutrient for optimal health. Iron has relevance for high-level athletes, contributing to the fundamental processes that affect physical performance, cognition, immunity, and recovery. Iron deficiency is prominent in athletes with a variety of mechanisms contributing to heightened iron losses (e.g., environmental factors, iron suppressing hormones, menstruation, and physical contact) [1,2,3]. However, the severity of iron deficiency largely dictates the level of impairment in physical performance. For example, clinically low iron stores (serum ferritin (sFer) < 30 µg/L; hemoglobin > 120 g/L) negatively affect an individual’s ability to produce energy aerobically, consequently increasing the reliance on anaerobic metabolism [4]. The impeded ability to produce energy aerobically negatively impacts on performance through heightened blood lactate and energy requirements compared with an iron-sufficient athlete [4,5,6,7]. When hemoglobin (Hb) concentrations are also reduced (<120 g/L), an individual becomes anemic, resulting in a reduced ability to deliver oxygen to the working muscles, as well as a limited ability to produce new red blood cells. Therefore, compromised iron stores may lead to negative outcomes for athletes in training and competition.

Iron requirements are greater for females to compensate for the additional losses experienced from sex-specific mechanisms (e.g., the suppression of estrogen, losses from menses) [8]. For instance, ~20–30% more female triathletes and long-distance runners are ID (~60% overall) than males [9]. Given the high prevalence of iron deficiency in aerobic-dominant athletes and the biochemical processes of iron that are commonly associated with the physical determinants of successful aerobic performance, most research is centered around this population. For example, time-trial performance has been impaired by up to ~20% [4,5,6,7] in clinically iron-deficient (ID) non-anemic female athletes (sFer < 30 µg/L). However, the prevalence of iron deficiency is equally high in female team sport athletes with up to 60% of female team sport athletes also presenting with iron deficiency [10,11,12]. The negative effects of iron deficiency are likely to be as detrimental to team sport athletes as aerobic-dominant athletes. Due to the lack of literature on team sport athletes, practitioners working in these settings are basing their recommendations on athletes outside their sport.

A successful physical performance in team sport is typically characterized by high-levels of strength, power, and speed. The biochemical processes of iron, including oxidative phosphorylation of adenosine diphosphate to adenosine triphosphate, and oxygen transportation and uptake, are central to many of the physical attributes characterizing a successful sporting performance. It is therefore imperative to understand if the other physical attributes that are necessary for team sport athletes are affected to a similar extent as endurance performance and maximal aerobic capacity.

Australian Rules Football is an invasive team sport with sport-specific actions requiring well-developed physical attributes, such as endurance, strength, power, and speed [13,14,15]. These physical attributes are necessary in movements such as a player accelerating and sprinting over short distances for chase-down tackles, or the power and strength displayed when players are required to tackle and jump to out-mark their opponent when competing for the ball. It is therefore plausible that iron status may affect Australian Football League Women’s (AFLW) players’ training and match performance. The aim of this study was to explore the effects of iron status on AFLW strength and power performance over a 10-week preseason training phase.

## 2. Materials and Methods

### 2.1. Experimental Design and Subjects

This study followed a 10-week exploratory, prospective, longitudinal (observational) design during the 2024 AFLW preseason, examining the associations between iron status and strength and power performance in elite AFLW players. Thirty female players (age: 24 ± 4 y, height: 1.7 ± 0.1 m, body mass: 76 ± 11 kg; mean ± SD) competing in the AFLW from the same team participated. All the players were informed of the study design, risk, and benefits prior to providing their written informed consent. This study was approved by the University of Canberra Human Research Ethics Committee (approval number 12213).

### 2.2. Methodology

Blood samples (~10 mL) were collected using venipuncture from a forearm vein at the start (week 1) and end (week 10) of the 2024 AFLW preseason to monitor the iron status. A total of 27 players had both venous samples collected, and two had one sample collected. Additionally, in week 9, another player withdrew from the study for personal reasons. Blood samples were collected at the same time of day (1230–1500 h) following at least 24 h of no physical activity. At the time of testing, no players were sick or injured.

Players were categorized as either ID (sFer <40 µg/L) or iron sufficient (IS; sFer ≥ 40 µg/L). An sFer threshold of <40 µg/L was utilized given that female athletes have demonstrated greater iron losses throughout heightened periods of activity (e.g., preseason) and this reflects similar sFer thresholds implemented in other female athletic cohorts to categorize iron deficiency (non-anemic iron deficiency stage 1) [10,16,17,18]. It is important to note that two players, identified as having non-anemic iron deficiency stage 2 (sFer < 20 µg/L and hemoglobin concentration > 120 g/L) following the blood samples in week 1, received an iron infusion (10 mL, 500 mg elemental iron) from the team’s sports physician. No player was identified as having iron-deficiency anemia (sFer < 12 µg/L, Hb < 120 g/L).

To evaluate the strength and power performance, a gymnasium and field test battery were conducted during week 1 and week 10 at the same time of day. The test battery was designed by the team’s high-performance manager in accordance with routine practice in Australian Football and other team sports [19,20]. Specifically, the test battery included a three-repetition maximum bench press, squat, and hip thrust; counter-movement jump (CMJ), 10-m sprint, and maximal velocity achieved during a 40-m sprint. The compound strength assessment (squat, bench press, and hip thrust) and CMJ performance were recorded during gymnasium sessions throughout week 1 on alternate days. Following a general warm-up implemented by the high-performance manager, players completed repetitions at an increasing percentage of their estimated 3RM. Specifically, players completed 10 repetitions at 50%, 6 repetitions at 60%, 5 repetitions at 70%, 5 repetitions at 80%, and 3 repetitions at 90%, with at least a 2-min recovery between sets. Following this structured warm-up, players performed 3RM lifts until failure with at least 3 min between sets. The CMJ data were collected using Force Decks (Vald Performance, Newstead, QLD, Australia) with players completing three maximal jumps with 10 s between efforts. The CMJ performance was measured using the highest power generated across the three jumps, and this was used for the analysis. The bench press, squat, hip thrust, and CMJ power results were standardized to each player’s body mass.

10-m sprint and maximal velocity were recorded during the field training sessions following a general warm-up implemented by the high-performance manager. Players completed three 10-m sprint efforts from a stationary start with at least a 2-min recovery between efforts. The 10-m sprint data were recorded using the SmartSpeed timing gate system (Vald Performance, Newstead, QLD, Australia). The maximal velocity was recorded during the 40-m sprint efforts. The maximal velocity was obtained from the Catapult Vector S7 GPS unit (Catapult Sports, Melbourne, Australia) with the reliability and validity of these devices previously reported [21].

### 2.3. Statistical Analysis

Descriptive statistics for iron indices and physical assessment are presented as mean ± SD. Effect sizes with 90% confidence intervals were calculated to interpret the differences between ID and IS players: <0.20 (trivial), 0.20 to 0.59 (small), 0.60 to 1.19 (moderate), 1.20 to 1.99 (large), and >2.00 (very large). Linear mixed-effects models were utilized to assess the influence of sFer on each performance assessment. sFer and week were included as fixed effects for all the linear models. Relative squat was included as a fixed effect for CMJ power, maximal velocity, and 10-m sprint; hip thrust was included as a fixed effect for CMJ power; and CMJ power was included as a fixed effect for maximal velocity and 10-m sprint. For every linear model, athlete was included as a random effect. The models were fitted using the ‘lmer’ function from the ‘lme4’ package (version 1.1-35.3), utilizing the restricted maximum likelihood estimation (ReML) method in RStudio version 4.3.1. All the variables in the linear models for maximal velocity and 10-m sprint were standardized (z-scored) to permit between-player comparisons. The model estimates reflect the change in the performance variable (in standard deviations) for each one-standard-deviation increase in a moderating variable (fixed or random). The models were specified as follows:

1. Bench press, squat, and hip thrust
  lmer(Response ~ 1 + sFer × Week + (1|Athlete), data = data_source)
2. CMJ power
  lmer(Response ~ 1 + sFer × squat_rel + sFer × hipthrust_rel + Week + (1|Athlete), data = data_source)
3. Maximal velocity and 10-m sprint
  lmer(scale(Response) ~ 1 + scale(sFer) × scale(squat_rel) + scale(sFer) × scale(CMJ_rel) + Week + (1|Athlete), data = data_source)

Graphics were developed using the ggplot2 package (version 3.5.1). Estimated marginal means were fitted with the ‘emmip’ function utilizing 95% CIs. Pearson’s correlation values were interpreted as <0.20 (very weak), 0.20–0.39 (weak), 0.40–0.59 (moderate), 0.60–0.79 (strong), and ≥0.80 (very strong). Goodness of fit was assessed through a residual analysis, with the statistical significance set at *p* < 0.05.

## 3. Results

The prevalence of iron deficiency increased from ~47% at the start of the preseason (week 1) to 52% at the end of the preseason (~52%) with similar overall team’s mean sFer for both weeks (week 1: 44 ± 22 µg/L; week 10: 48 ± 49 µg/L). Mean ± SD values for all the iron indices for both groups at the start and end of the preseason are provided in Table 1. The mean change in iron indices between both timepoints for all the players was trivial (effect size < 0.20) to small (0.50). The mean (± SD) values for all the iron indices for the team are presented in the online Supplementary File S1: Table S1.

The effect size analysis showed a very large difference in sFer concentration in favor of the IS group at both the start (*d* = −2.4; 90% CI −3.2 to −1.6; Table 1) and end of the preseason (*d* = −3.4; 90% CI −4.4 to −2.3). There was a difference in Hb during week 1 (*d* = 0.4; 90% CI −0.2 to 1.0; *p* = 0.24) in favor of ID players, while non-significant trivial to small differences were evident for all the remaining iron indices in favor of IS players across both weeks. The three physical assessments (CMJ relative power, maximal velocity, and 10-m sprint) showed trivial differences in week 1 (Table 2). Hip thrust strength was moderately higher (*d* = −0.6; 90% CI −1.3 to 0.1), while bench press (*d* = −0.5; 90% CI −1.2 to 0.1) and squat (*d*= −0.5; 90% CI −1.2 to 0.2) strength demonstrated a moderate difference, with IS players outperforming ID players. The difference (4%) in hip thrust strength remained small (*d* = −0.3; 90% CI −1.1 to 0.4) in week 10 with IS players again outperforming ID players, while ID players recorded greater CMJ relative power results (small; *d* = −0.0; 90% CIs −0.6 to 0.) than IS players. There were variable differences in favor of both groups for all remaining strength and power assessments.

**Figure 1:**
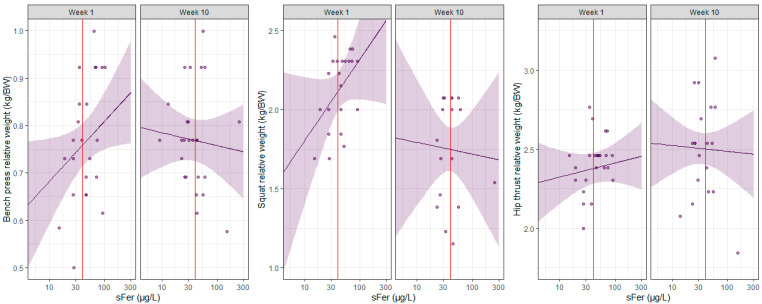
Scatterplot of the relative bench press, squat, and hip thrust strength for each individual player plotted against serum ferritin (sFer), separated by week. The purple line represents the predicted relative weight lifted by the players with, with 95% CIs in purple. The vertical red line indicates an sFer of 40 µg/L, the diagnostic cut-off used in the current study to categorize iron deficiency. The ReML analysis indicated no meaningful effect of sFer on any strength measure on a linear scale. There were modest effects of sFer on bench press (0.1 kg/BW; 95% CI 0.0 to 0.1) and squat (0.2 kg/BW; 95% CI 0.0 to 0.5) on a logarithmic scale. Marginal r-squared values indicated very weak to moderate correlations between each strength assessment and the moderating variables (i.e., sFer and week). Conditional r-squared values indicated weak to very strong associations when accounting for the random effect (athlete).

**Figure 2:**
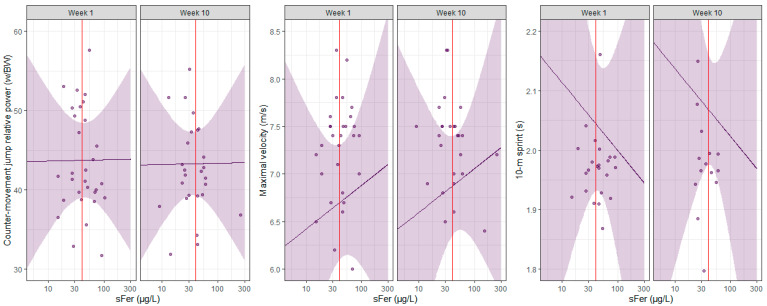
Scatterplot of counter-movement jump relative power, maximal velocity, and 10-m sprint performance for each individual player plotted against serum ferritin (sFer), separated by week. The purple line represents the predicted performance by the players, with 95% CIs in purple. The vertical red line indicates an sFer of 40 µg/L, the diagnostic cut-off used in the current study to categorize iron deficiency. The ReML analysis indicated a modest effect of sFer on all the power and speed measures. There was an estimated improvement of 0.2 W/BW (95% CI: −1.0 to 1.4) for counter-movement jump performance for every one-unit increment in sFer. Similarly, sFer had a predicted effect of 0.2 m/s (95% CI: −0.3 to 0.8) on maximal velocity and −0.4 s (95% CI: −1.1 to 0.4) on 10-m sprint performance. Marginal r-squared values indicated very weak to moderate correlations between each strength assessment and the moderating variables (i.e., sFer, week, hip thrust, squat, counter-movement jump relative power). Conditional r-squared values indicated weak to very strong associations when accounting for the random effect (athlete).

Marginal r-squared values ranged between 0.0 and 0.4 indicating very weak to moderate correlations between each strength and power assessment and all the moderating variables (e.g., sFer, week, squat). Conditional r-squared values increased from 0.3 to 0.9, indicating weak to very strong associations when accounting for the random effect (athlete) as well as the fixed effects. The results of each ReML model are provided in Table 3 and Table 4 and visualized in Figure 1 and Figure 2.

## 4. Discussion

The sFer concentration ranged between 9 and 102 µg/L across both time points (excluding the two venous samples in week 10 of both players who received an iron infusion following week 1). Approximately 80% of all the samples showed an sFer concentration of <60 µg/L. Iron status had a highly variable association with all the strength and power assessments at the start and end of the preseason phase. Impairment in strength performance (bench press, squat, hip thrust) appeared to coincide with greater differences in sFer between players. However, a low iron status (i.e., sFer) may not translate directly to sport-specific measures of power and speed, including CMJ power, maximal velocity, and the 10-m sprint. Iron monitoring practices in team sports should be employed to identify the individual thresholds at which athlete health and performance may be compromised to negate any negative effects on training and competition.

The consequences of an impaired iron status on strength performance in high-level athletes remains uncertain. The same mechanism by which iron facilitates maximal aerobic capacity could underpin the fluctuations in strength performance in athletes with a low sFer status [22]. That is, despite single sets of strength performance not being directly dependent on sufficient iron indices, reduced Hb may limit the tissue oxygenation of the working skeletal muscle between sets. A decline in tissue oxygenation may negatively affect recovery, reducing the total training volume and potentially any of the subsequent skeletal muscle adaptation to the desired strength stimulus. Our findings lend support to this proposed mechanism given that ID players did not display an impaired strength and power performance relative to IS players across 75% of the assessments. This outcome may relate to the ID group exhibiting a mean Hb concentration above the threshold adopted to categorize an iron-deficiency anemia individual (i.e., Hb < 120 g/L). Therefore, the ID group were unlikely to experience any impairment in tissue oxygenation to negatively affect performance. However, the results may be masked by the similarity in iron status between the two groups. We note a superior sFer concentration of 25–35 µg/L higher in the IS group at both time points relative to the sFer status of the ID group. Further, a binary threshold dividing players into two groups could be limited in determining whether an improved iron status coincides with an improved strength performance.

The threshold implemented to establish ID and IS players may have been inadequate to examine decisively the differences in performance. Indeed, the criteria utilized for the IS group were still inclusive of players who were functionally ID (sFer < 100 µg/L) [22,23]. This point is of particular relevance as functional iron deficiency is characterized as individuals with adequate ferritin stores, but a relative inability to mobilize and utilize iron. This can result in negative sports performance outcomes similar to those athletes with low ferritin stores (e.g., reduced capacity to generate energy aerobically) [24]. This sequence can occur in response to inflammation and heightened hepcidin levels, which block the solitary cellular iron exporter ferroportin [25] in response to increased training loads, such as those experienced typically throughout the preseason periods. In the current team, only three players had an sFer status of >100 µg/L that is representative of iron normal at either the start or end of the preseason. Importantly, bench press strength was impeded or showed no change (−11 to 0%) in association with an sFer of <100 µg/L, while the response to higher iron stores on CMJ power and maximal velocity was either highly variable or absent (−4 to +5%). However, lower body strength can also decline in response to an increase in fatigue and heightened training loads (e.g., preseason periods), which may account for the highly variable response on lower body assessments [26,27,28,29]. For example, CMJ height and power substantially reduced in association with fatigue (r ≤ −0.742) in male collegiate hockey players [28]. It is possible that an increase in training loads may have resulted in higher fatigue scores in the AFLW players, nullifying any difference between ID and IS players that otherwise may have been anticipated in week 10. It is difficult to discern whether the players would benefit from an improved iron status in the current team considering the uniform low iron status across the group. It is plausible for strength and power performance to improve in both groups of players (ID and IS), given that players with a functional iron deficiency may be compromised to the same extent as those identified as ID with a low sFer status. Therefore, additional seasonal iron monitoring would be useful to identify the individual thresholds at which strength performance may be impaired in each athlete.

In female athletic cohorts, even ID athletes with normal Hb have shown that strength performance may be impaired. For example, isokinetic strength performance was up to 23% lower across 80% of assessments in ID endurance athletes compared to their IS counterparts [30]. Strength performance also improved following iron intervention in elite ID female volleyball players providing further evidence that strength performance in ID athletes may be reduced irrespective of their Hb. Despite only four players categorized as clinically ID, strength performance (power clean, clean and jerk, total mean strength) improved by up to 45% following iron supplementation (~105 mg/d elemental iron; iron supplementation group mean sFer declined by ~8%) [22]. The results of the linear models lend support this notion indicating the degree to which strength performance is limited is dictated by the severity of compromised sFer stores, not Hb.

When comparing strength (bench press, squat, hip thrust) for an IS player with an sFer of 100 µg/L with an ID player with an sFer of 10 µg/L, the modelled performance is anticipated to be up to 15% greater in the IS player. This view also becomes evident when comparing groups with a strength performance that is impeded to a greater extent when the difference in iron status was greater. Specifically, IS players recorded strength results of up to 13% higher than ID players, while also recording a mean sFer status of 35 µg/L higher. However, by the end of the preseason when there was a difference in the sFer status of 25 µg/L, hip thrust was 4% lower in ID players, but there was no difference in bench press or squat. These results highlight the potential strength decrements that ID athletes may be predisposed to irrespective of Hb concentration. Further, these results indicate that other mechanisms beyond a reduction in tissue oxygenation likely underpin the reductions in strength performance in IDNA athletes.

The reduced strength performance in IDNA athletes may be explained by heightened adenosine monophosphate-activated protein kinase (AMPK) activity; however, this mechanism warrants further research. AMPK regulates energy expenditure and inhibits protein synthesis [31], with iron deficiency augmenting AMPK activity in response to energetic stress (e.g., reduced oxygen supply). Consequently, heightened AMPK activity shifts the metabolism towards anaerobic energy pathways (i.e., phosphocreatine), subsequently exacerbating muscular fatigue [31]. Furthermore, iron deficiency may alter the AMPK subunit composition, increasing AMPKα1 activity (inhibits protein synthesis) and reducing AMPKα2 content (supports ATP production). Given that AMPKα1 inhibits protein synthesis and promotes glycolytic metabolism, this mechanism may underpin the strength decrements in ID athletes, negatively affecting adaptation, hypertrophy, and overall strength outcomes.

While the direct effect of AMPK activity on strength adaptation in athletes remains unclear, prolonged iron deficiency has been linked to chronic AMPK activation [31]. ID athletes may therefore be predisposed to unfavorable strength adaptation outcomes considering the role that AMPKα1 plays in the inhibition of protein synthesis. However, any potential longitudinal effect of elevated AMPK seems to be inferior compared with heightened training loads, given that there was no difference in strength outcomes between ID and IS players by the end of the preseason. It was not possible to record any direct or proxy measure of AMPK activity in the current study. Therefore, it is important to acknowledge that the proposed mechanism of AMPK underpinning impaired strength in ID players is only speculative. It would be beneficial for future confirmatory mechanistic research to investigate direct or proxy measures of AMPK activation and signaling pathways mediating strength decrements in ID human participants (e.g., via muscle biopsies), particularly comparing ID participants with IS participants who are considered iron normal (>100 µg/L).

The importance of AMPK in underpinning strength decrements in ID athletes may extend to sport-specific power and speed measures. Given the heightened dependence on phosphocreatine and increased muscular fatigue, it is reasonable to anticipate that power and speed performance (e.g., CMJ, 10-m sprint, maximal velocity) might also be impeded in ID players. Furthermore, the transfer of strength from movements including a squat and hip thrust to power and speed attributes, including jumping, sprinting, and accelerating, is well documented [20,32,33]. Therefore, ID athletes may be predisposed to a compounding negative effect whereby low iron stores directly affect movements that are reliant on power and speed and limit strength adaptation, thereby attenuating transfer between movements (e.g., squat to CMJ).

While the implications of iron deficiency were more apparent on strength (i.e., bench press, squat, hip thrust), the implications of iron deficiency on power and speed were less conclusive. It is plausible that the differing testing protocols employed may have contributed to the more modest influence of sFer. That is, the strength assessments were preceded by structured warm-up protocols consisting of a decreasing repetition scheme with an increase in weight leading up to the 3RM attempts. It is also important to note that there was no maximum limit of attempts; therefore, some players completed the assessment after three attempts while others completed the assessment after five attempts. Comparatively, the protocol for CMJ, 10-m sprint, and maximal velocity consisted of three singular maximal efforts interspersed with a recovery period. It may be likely that the protocol for the strength assessments has induced a greater effect of fatigue with unequal testing volumes, which is relevant for iron-deficient athletes if there is indeed a greater reliance on phosphocreatine [31]. This is compared with the protocol of the power and speed assessments where all the players completed the same number of attempts, thus there was no disparity in testing volume. Nevertheless, the effect of sFer on speed and power is projected to increase with greater iron stores.

Utilizing the mean sFer status of both groups at week one (ID: sFer 28 µg/L; IS: sFer 63 µg/L), sFer accounted for the largest difference in CMJ (14%). Further, there was a 4% improvement for every one-unit increment in the hip thrust relative weight (e.g., 1 × body weight to 2 × body weight) and no effect of squat. The limited transfer to the squat is difficult to reconcile given the established strong relationship between squat and CMJ performance [33]. Similar results from the linear models were evident with maximal velocity and 10-m sprint performance, whereby sFer had a larger effect than squat. Specifically, a one-standard-deviation change in the sFer status was predicted to affect maximal velocity and 10-m sprint by 2% and −1%, respectively, suggesting that marked differences in performance may become more evident with larger differences in iron status. For example, comparing the sFer status’ three standard deviations apart for maximal velocity accounted for a 5% difference in performance (i.e., 7.18 m/s compared with 7.55 m/s).

Importantly, these estimates should be interpreted with caution given the small sample size and wide confidence intervals (e.g., −8.4 to 8.1 for squat as a fixed effect for the CMJ predictions). It remains unclear whether the null effect of squat on CMJ, 10-m sprint, and maximal velocity represents a genuine absence of effect, or whether the analysis was underpowered. Given the known link between squat and CMJ performance, the latter seems plausible. While the transfer of strength assessments (i.e., squat, hip thrust) to sport-specific power and speed measures (i.e., CMJ power, maximal velocity, 10-m sprint) was modest (−1 to +4% for each unit or standard deviation increment), it remains that low iron stores may have a negative effect on power and speed, with the severity of ID coinciding with performance outcomes. These preliminary observations highlight the need for more extensive research to examine athletes with a broader spectrum of iron statuses. Iron status in the current team was heavily skewed towards an ID status, potentially masking any effect that may be elicited if a team comprising a broader spectrum of sFer statuses were examined.

### 4.1. Limitations

It is important to acknowledge that given that this study was conducted in an applied professional setting, there are inherent strengths but also some limitations. Notably, this research was exploratory in nature, utilizing convenience sampling without a priori power analysis. Subsequently, the sample size was naturally small for the subgroup analysis but was further reduced in some assessments, particularly in week 10, due to player unavailability (e.g., injury, personal reasons) and issues with club equipment. Consequently, the small sample size reduced the statistical power, contributing to wide confidence intervals and increased risks of type II errors. The wide confidence intervals limit the precision of the findings, making it more difficult to ascertain with confidence the direct effect of sFer on the assessed strength and power measures. This limitation should therefore be considered when interpreting the results, with the findings considered as exploratory and hypothesis-generating, and not confirmatory in nature [34]. It would be beneficial for future research to pool cohort data or conduct research on larger athletic populations to provide a more accurate estimate of the effect(s) of iron status on strength performance measures.

Secondly, lower body strength and power may decline in response to increased training loads, such as those experienced during the preseason. It is possible that an increase in training loads may have resulted in higher fatigue scores in the AFLW players, nullifying any difference between the ID and IS players that otherwise may have been anticipated in week 10. Wellness data (e.g., soreness and readiness) were collected throughout the study with the intention of including these data in the linear models. However, the majority (66–82%) of responses were skewed towards a single answer (66–82%) and combined with the second-most popular response on each question, accounted for 81–97% of all the data, reflecting the same challenge that was previously observed in collecting wellness data in elite athletes [35,36]. Given the limited variability, these measures were excluded from the linear models as they provided insufficient information to meaningfully contribute to the analysis.

Finally, utilizing a binary threshold to assess the differences between ID and IS has limitations, particularly during periods of heightened activity (e.g., preseason) when fluctuations in individual iron status are evident. Monitoring the iron status longitudinally using individual thresholds at which the overall health and sports performance may be compromised [23] would be informative. However, female athletes and teams are often financially limited, with teams frequently employing part-time staff with a high turnover [37]. It remains difficult for elite female teams to implement evidence-based best practice when facing these constraints and instability in staff support.

### 4.2. Practical Implications

Iron deficiency trended towards negatively affecting strength performance (bench press, squat, hip thrust) by up to 13%, with impairments in strength and power predicted to increase with greater differences in sFer. It therefore seems pertinent to implement longitudinal iron monitoring practices in elite female teams given that approximately half of the team presented as ID at both the start and end of the preseason. However, it must also be considered that iron monitoring practices will largely differ based on a variety of factors, including the type of sport, training loads, individual history, diet, and financial constraints. The frequency of screening may also affect the thresholds utilized to categorize and treat iron deficiency among different sport teams. For example, individualized thresholds are encouraged when bi-annual or quarterly screening frequencies are feasible, with the first sample collected at the start of the preseason used as a benchmark. Alternatively, at a minimum, higher binary thresholds to categorize non-anemic iron deficiency stage 1 (e.g., sFer < 40 µg/L) may be used. However, if teams are financially constrained to one screening per season, higher thresholds (e.g., sFer < 100 µg/L) may be utilized as a pre-cautionary measure to cater for the large sFer deficits that female athletes may encounter. A higher sFer cut-off (e.g., sFer < 100 µg/L) also accounts for athletes who may present as functionally ID with a sports performance that is negatively affected similarly to those athletes classified as having non-anemic iron deficiency stage 1.

If there are no financial or logistical constraints, teams are encouraged to screen athletes bi-annually relative to the season length, including the first screening at the start of pre-season. The first sample at the start of the preseason may be used as a benchmark for each individual, when the athletes are presumably returning to training and have had a period of lower activity allowing iron stores to replete. For athletes identified as ID, the frequency of monitoring should increase to quarterly and, again, this may be made relative to the season length. Adjusting the frequency relative to the season length will allow practitioners to monitor ID athletes throughout different phases of the season (e.g., preseason, in-season, post-season), as well as identify individual responses to iron supplementation that can be manipulated based on changes in iron status and related clinical considerations.

In the current study, no supplementation was provided to any player categorized as stage 1 iron-deficient non-anemic (sFer < 40 µg/L, Hb > 120 g/L) in week 1; only iron infusions in the two stage 2 iron-deficient non-anemic players. However, the players did not receive oral supplementation due to management and logistical constraints, rather than the team’s desired protocol. Considering the importance of strength, power, and speed in sport-specific actions, including chase-down tackles, tackling, and jumping to out-mark opponents, it is imperative that iron treatment protocols (e.g., 100 mg/d or bi-daily of elemental iron) are implemented to enhance overall athlete health and sports performance.

## 5. Conclusions

Low iron stores may compromise strength performance (bench press, squat, hip thrust) in elite iron-deficient female football players, but the impairments may not transfer directly to measures of power and speed. All the potential effects of iron status raised in this study need to be confirmed in future confirmatory research with an adequate error control. It is important for teams to implement longitudinal iron monitoring and intervention strategies to minimize the number of athletes with low iron stores.

## Figures and Tables

**Table 1:** Iron indices (mean ± SD) recorded across the 2024 AFLW preseason between iron-deficient (ID; sFer < 40 µg/L) and iron-sufficient (IS; sFer ≥ 40 µg/L) players and effect size comparisons.

Start of Preseason (Week 1)				
Iron Measure	ID (n = 14)	IS (n = 16)	Cohen’s *d* (90% CI)	*p* Value
	Mean (SD)	Mean (SD)	ID:IS	
sFer (µg/L)	28 ± 8	63 ± 19	−2.4 (−3.2 to −1.6)	<0.00
Hb (g/L)	133 ± 6	130 ± 6	0.4 (−0.2 to 1)	0.24
MCV (fl)	92 ± 3	92 ± 4	0 (−0.6 to 0.6)	0.76
MCHb (pg)	30 ± 1	31 ± 1	−0.2 (−0.8 to 0.4)	0.67
**End of Preseason (Week 10)**				
**Iron Measure**	**ID (n = 14)**	**IS (n = 13)**	**Cohen’s *d* (90% CI)**	***p* Value**
	**Mean (SD)**	**Mean (SD)**	**ID:IS**	
sFer (µg/L)	25 ± 8	50 ± 7	−3.4 (−4.4 to −2.3)	<0.00
Hb (g/L)	137 ± 8	144 ± 27	−0.4 (−1.0 to 0.3)	0.94
MCV (fl)	92 ± 4	93 ± 3	−0.3 (−1.0 to 0.3)	0.54
MCHb (pg)	30 ± 1	31 ± 1	−0.5 (−1.1 to 0.2)	0.24

AFLW Australian Football League Women’s, sFer serum ferritin (clinically normal range = 30–300 ug/L), Hb hemoglobin concentration (normal range ≥ 120 g/L), MCV Mean cellular volume (normal range = 80–100 fl), MCHb Mean cellular hemoglobin (normal range = 27–33 pg).

**Table 2:** Relative physical performance results (excluding maximal velocity and 10-m sprint; mean ± SD) recorded across the 2024 AFLW preseason between iron-deficient (ID) and iron-sufficient (IS) players and effect size (Cohen’s *d*) comparisons.

Start of Preseason (Week 1)					
	Group	Sample	Mean (SD)	Cohen’s *d* (90% CI)	*p* Value
Bench press (kg/BW)	ID	10	0.7 ± 0.1	−0.5 (−1.2 to 0.2)	0.34
	IS	13	0.8 ± 0.1		
Squat (kg/BW)	ID	9	2.1 ± 0.3	−0.5 (−1.2 to 0.2)	0.29
	IS	13	2.2 ± 0.2		
Hip thrust (kg/BW)	ID	11	2.4 ± 0.2	−0.6 (−1.3 to 0.1)	0.12
	IS	13	2.5 ± 0.1		
CMJ power (W/BW)	ID	14	44 ± 7	0.1 (−0.5 to 0.7)	0.70
	IS	16	43 ± 7		
Maximal velocity (m/s)	ID	14	7.2 ± 0.5	−0.1 (−0.7 to 0.6)	0.65
	IS	15	7.3 ± 0.6		
10-m sprint (s)	ID	9	2.0 ± 0.0	0.0 (−0.7 to 0.7)	0.83
	IS	14	2.0 ± 0.1		
**End of Preseason (Week 10)**					
	**Group**	**Sample**	**Mean (SD)**	**Cohen’s *d* (90% CI)**	***p* Value**
Bench press (kg/BW)	ID	16	0.8 ± 0.1	0.0 (−0.7 to 0.6)	0.83
	IS	11	0.8 ± 0.1		
Squat (kg/BW)	ID	11	1.8 ± 0.3	0.0 (−0.7 to 0.8)	1.00
	IS	9	1.8 ± 0.3		
Hip thrust (kg/BW)	ID	14	2.5 ± 0.3	−0.3 (−1.1 to 0.4)	0.76
	IS	8	2.6 ± 0.3		
CMJ power (W/BW)	ID	15	41 ± 13	0.0 (−0.6 to 0.7)	0.40
	IS	12	41 ± 5		
Maximal velocity (m/s)	ID	17	7.4 ± 0.5	0.3 (−0.3 to 0.9)	0.35
	IS	12	7.2 ± 0.3		
10-m sprint (s)	ID	11	2.0 ± 0.1	0.2 (−0.7 to 1)	0.91

AFLW Australian Football League Women’s, CMJ counter-movement jump.

**Table 3:** Relative three-repetition maximum bench press, squat, and hip thrust estimates from the ReML analysis based on the interactions of fixed (sFer/log(sFer) and week), and random (athlete) effects. The linear scale reflects changes in fixed and random effects for every one-unit increment in sFer (i.e., 1 µg/L). Log-transformed sFer results are presented for a more meaningful interpretation of the changes across a wider range of sFer (e.g., 10 µg/L compared with 100 µg/L).

	Strength and Power Measure (Response Measure)					
	Linear Scale			Log Scale		
	Bench Press (kg/BW)	Squat (kg/BW)	Hip Thrust (kg/BW)	Bench Press (kg/BW)	Squat (kg/BW)	Hip Thrust (kg/BW)
Mean	0.8	2.0	2.5			
SD	0.1	0.3	0.2			
Intercept	0.7 (0.6 to 0.8)	1.9 (1.6 to 2.2)	2.4 (2.2 to 2.5)	0.6 (0.4 to 0.7)	1.3 (0.3 to 2.3)	2.2 (1.9 to 2.6)
sFer	0 (0 to 0)	0 (0 to 0)	0 (0 to 0)			
log(sFer)				0.1 (0.0 to 0.1)	0.2 (0.0 to 0.5)	0.0 (0.0 to 0.1)
Week 1	0	0	0	0	0	0
Week 10	0.1 (0.0 to 0.1)	−0.1 (−0.5 to 0.2)	0.2 (0.1 to 0.3)	0.3 (0 to 0.5)	0.6 (−0.8 to 1.9)	0.3 (−0.1 to 0.7)
sFer: Week 10	0 (0 to 0)	0 (0 to 0)	0 (0 to 0)			
log(sFer): Week 10				−0.1 (−0.1 to 0.0)	−0.3 (−0.6 to 0.1)	−0.1 (−0.2 to 0.0)
R2m/R2c	0.0/0.9	0.3/0.4	0.1/1	0.0/0.9	0.4/0.4	0.1/1

sFer: serum ferritin. Note: Week 1 is used as the reference week. R2m indicates the variance explained by the fixed effects. R2c indicates the variance explained by both the fixed and random effects.

**Table 4:** Counter-movement jump relative power, maximal velocity, and 10-m sprint estimates from the ReML analysis on a linear scale based on the interactions of fixed (sFer, relative squat, and week), and random (athlete) effects. Relative hip thrust has also been included as a fixed effect for counter-movement jump (CMJ) relative power estimations. Relative CMJ relative power has been included as a fixed effect for maximal velocity and 10-m sprint time estimations. All the variables in the linear models for maximal velocity and 10-m sprint (excluding week) were standardized (z-scored) to allow for comparability.

	CMJ Relative Power (W/BW)		Max Velocity (m/s)	10-m Sprint (s)
Mean	42	Mean	7.3	2.0
SD	8	SD	0.5	0.1
Intercept	40 (−16 to 96)	Intercept	−0.2 (−0.7 to 0.3)	0 (−0.5 to 0.5)
sFer	0.2 (−1.0 to 1.4)	scale (sFer)	0.2 (−0.3 to 0.8)	−0.4 (−1.1 to 0.4)
Squat	−0.1 (−8.4 to 8.1)	scale (Squat)	0.2 (−0.1 to 0.5)	0.3 (−0.1 to 0.8)
Hip thrust	2 (−22 to 26)	scale (CMJ power)	0.5 (−0.1 to 0.9)	−0.8 (−1.3 to −0.3)
Week 1	0	Week 1	0	0
Week 10	−0.3 (−3.7 to 3.1)	Week 10	0.4 (−0.2 to 1.0)	0.3 (−0.6 to 1.2)
sFer:squat	0.0 (−0.2 to 0.2)	scale (sFer:squat)	0.3 (−0.1 to 0.7)	−0.2 (−1.1 to 0.6)
sFer:hip thrust	−0.1 (−0.6 to 0.5)	scale (sFer:CMJ power)	−0.2 (−1 to 0.6)	0.3 (−0.8 to 1.4)
R2m/R2c	0.0/0.9	R2m/R2c	0.1/0.7	0.3/0.3

sFer: serum ferritin. Note: Week 1 is used as the reference week. R2m indicates the variance explained by the fixed effects. R2c indicates the variance explained by both the fixed and random effects.

## Data Availability

The data presented in this study are available on request from the corresponding author. The data are not publicly available due to ethical and privacy considerations.

## Supplementary Materials

The following supporting information can be downloaded at: https://www.mdpi.com/article/10.3390/nu17101691/s1, Table S1. Iron status and physical performance results (mean ± SD) recorded across the 2024 AFLW preseason and effect size comparisons between both time points.

## Abbreviations

The following abbreviations are used in this manuscript:

sFer	Serum ferritin
ID	Iron deficient
IS	Iron sufficient
Hb	Hemoglobin concentration
AFLW	Australian Football League Women’s
CMJ	Counter-movement jump
REML	Restricted maximum likelihood
