# Supplementary material for: Iron’s True Weight: Does the Amount of Iron in the Body Equate to the Amount of Iron on the Bar in Australian Football League Women’s Players?
Source: Nutrients. 2025 May 16;17(10):1691. doi: 10.3390/nu17101691 (PMC12114154; doi:10.3390/nu17101691)
Supplement: Supplementary file 1 [file nutrients-17-01691-s001.zip › nutrients-3597898-supplementary.pdf]

Table S1. Iron status and physical performance results (mean ± SD) recorded across the 2024 AFLW preseason and effect size comparisons between both time points.

	Start of preseason (Week 1)	End of preseason (Week 10)	Cohen's d (90% CIs)	
			Week 1:Week 10	p value
n=	30	27		
sFer (µg/L)	44 ± 22	48 ± 49	-0.1 (-0.4 to 0.2)	0.71
ID (n)	14	14		
Hb (g/L)	132 ± 6	140 ± 18	-0.4 (-0.8 to -0.1)	0.03
MCV (fl)	92 ± 3	92 ± 3	-0.1 (-0.4 to 0.3)	0.77
MCHb (pg)	30 ± 1	31 ± 1	-0.1 (-0.4 to 0.2)	0.51
Bench press (kg/BW)	0.8 ± 0.1	0.8 ± 0.1	-0.2 (-0.6 to 0.3)	0.59
Squat (kg/BW)	2.1 ± 0.2	1.8 ± 0.3	1.2 (0.6 to 1.7)	<0.00
Hip thrust (kg/BW)	2.4 ± 0.2	2.5 ± 0.3	-0.3 (-0.8 to 0.2)	0.33
CMJ power (w/BW)	43 ± 6	41 ± 10	0.3 (-0.1 to 0.7)	0.28
Maximal velocity (m/s)	7.3 ± 0.5	7.3 ± 0.4	-0.1 (-0.5 to 0.3)	0.67
10-m sprint (s)	2 ± 0.1	2 ± 0.1	-0.2 (-0.7 to 0.4)	0.65

sFer serum ferritin, *ID* iron deficient, *Hb* haemoglobin concentration, *H'crit* haematocrit, *MCV* Mean cellular volume, *MCHb* Mean cellular haemoglobin, *Vit D* vitamin D
